# Cyclin-Dependent Kinase Activity Controls the Onset of the HCMV Lytic Cycle

**DOI:** 10.1371/journal.ppat.1001096

**Published:** 2010-09-09

**Authors:** Martin Zydek, Christian Hagemeier, Lüder Wiebusch

**Affiliations:** 1 Children's Hospital, Laboratory for Molecular Biology, Charité Universitätsmedizin Berlin, Berlin, Germany; 2 Free University of Berlin, Faculty of Biology, Chemistry and Pharmacy, Berlin, Germany; University of Minnesota, United States of America

## Abstract

The onset of human cytomegalovirus (HCMV) lytic infection is strictly synchronized with the host cell cycle. Infected G0/G1 cells support viral immediate early (IE) gene expression and proceed to the G1/S boundary where they finally arrest. In contrast, S/G2 cells can be infected but effectively block IE gene expression and this inhibition is not relieved until host cells have divided and reentered G1. During latent infection IE gene expression is also inhibited, and for reactivation to occur this block to IE gene expression must be overcome. It is only poorly understood which viral and/or cellular activities maintain the block to cell cycle or latency-associated viral IE gene repression and whether the two mechanisms may be linked. Here, we show that the block to IE gene expression during S and G2 phase can be overcome by both genotoxic stress and chemical inhibitors of cellular DNA replication, pointing to the involvement of checkpoint-dependent signaling pathways in controlling IE gene repression. Checkpoint-dependent rescue of IE expression strictly requires p53 and in the absence of checkpoint activation is mimicked by proteasomal inhibition in a p53 dependent manner. Requirement for the cyclin dependent kinase (CDK) inhibitor p21 downstream of p53 suggests a pivotal role for CDKs in controlling IE gene repression in S/G2 and treatment of S/G2 cells with the CDK inhibitor roscovitine alleviates IE repression independently of p53. Importantly, CDK inhibiton also overcomes the block to IE expression during quiescent infection of NTera2 (NT2) cells. Thus, a timely block to CDK activity not only secures phase specificity of the cell cycle dependent HCMV IE gene expression program, but in addition plays a hitherto unrecognized role in preventing the establishment of a latent-like state.

## Author Summary

Cyclin-dependent kinases (CDKs) control the cell division cycle. Many viruses employ CDK activity to control critical steps of their own replication cycle and to synchronise their replication with the cell cycle dependent availability of vital cellular enzymes and molecular building blocks. Here we show an unexpected antiviral function of CDK activity at a very early stage of human cytomegalovirus (HCMV) infection, the onset of immediate early (IE) gene expression. HCMV is unique amongst herpesviruses in being unable to initiate IE gene expression during the S/G2 phase of the cell cycle. CDK inhibition by either DNA damage-dependent induction of the cellular CDK inhibitor p21 or by the pharmacological CDK inhibitor roscovitine overcomes this limitation and makes S/G2 cells fully permissive for HCMV. Importantly, in undifferentiated NTera2 (NT2) cells, which normally establish a quiescent, latent-like HCMV infection, CDK inhibition also relieves the block of IE gene expression, suggesting a more general role for CDK activity in the control of this important human pathogen.

## Introduction

Human cytomegalovirus (HCMV) is a wide-spread human pathogen causing serious disease in immunocompromised patients and neonates [1]. As with all herpesviruses, HCMV exists either in a latent, asymptomatic state or undergoes poductive replication leading to lysis of the host cell. Lytic replication starts with the onset of viral immediate early (IE) gene expression. IE gene products, especially the major IE (MIE) proteins IE1 and IE2, have essential functions in host cell regulation and in activating the subsequent cascade of viral early and late gene expression [2]. In latently infected cells, MIE gene transcription is silenced and consequently viral gene expression is restricted to only very few genomic loci [3], [4], [5], [6]. Reactivation from latency is achieved by mechanisms that trigger desilencing of the MIE promoter/enhancer [7], [8], [9]. Thus, control of MIE gene expression is pivotal to the outcome of infection and, therefore, represents a main focus of HCMV research. In addition, MIE gene expression as the initial step in HCMV replication is considered a prime target for antivirals and an IE2-specific antisense-RNA (fomivirsen) has already proven to be effective in the local treatment of HCMV retinitis [10].

Interestingly, latent infection is not the only situation where HCMV replication is blocked at the level of MIE gene expression. For primary fibroblasts it has been shown that the cell cycle state at the onset of infection determines whether viral gene expression is initiated or not. In G0/G1, IE gene expression starts immediately while in S/G2 phase, transcription of IE1 and IE2 is efficiently suppressed [11], [12]. However, infection of S/G2 fibroblasts does not fully prevent but rather delays the onset of the lytic cycle until cells have completed cell division and reentered the next G1 phase. The physiological relevance of the cell cycle dependent regulation of HCMV is not understood. Furthermore, it is unclear what makes S/G2 cells non-permissive for MIE gene expression and whether the underlying mechanism also plays a role in the establishment of HCMV latency.

Here we analyzed the molecular determinants of cell cycle dependent repression of HCMV major IE genes. We found that inhibition of cyclin dependent kinase activity either by checkpoint activation or the chemical inhibitor roscovitine was sufficient to fully restore virus permissiveness in S/G2. Moreover, CDK inhbition was also successful in antagonizing the silencing of lytic gene expression during quiescent, latent-like infection of undifferentiated NTera2 (NT2) cells, suggesting a mechanistical link between cell cycle and latency-associated repression of IE gene transcription.

## Results

### The cell cycle-dependent block to HCMV MIE gene expression is neither a strain nor a cell type-specific phenomenon

**Figure 1:**
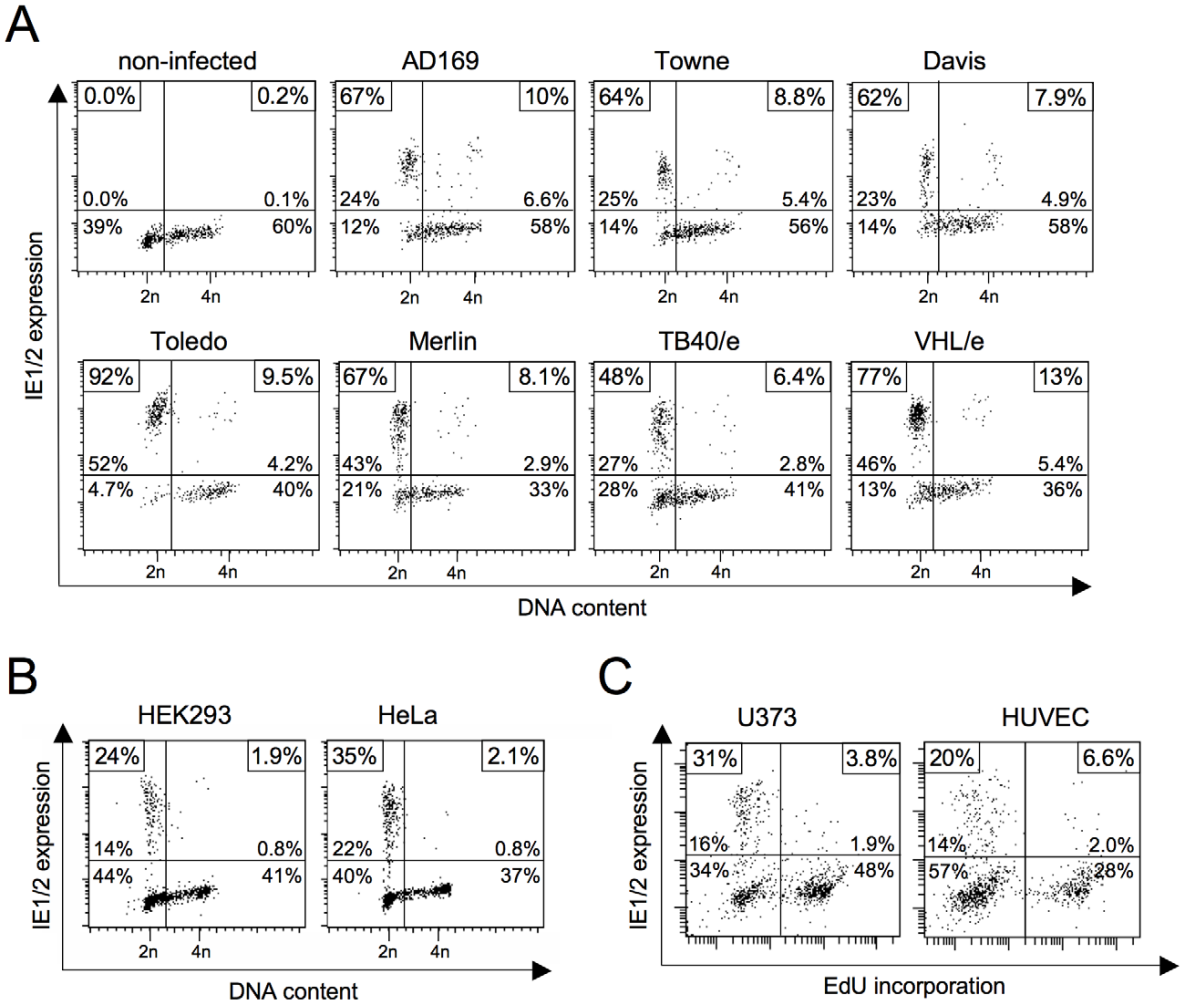
Block of HCMV major immediate early (MIE) gene expression in S/G2 is neither virus strain nor cell type-dependent. (A) Asynchronously proliferating HEL fibroblasts were infected with the indicated HCMV strains at high multiplicity of infection (MOI = 5). Three hours post infection (hpi) cells were harvested and analyzed for DNA content and MIE gene expression by flow cytometry. Shown are dot plots where cells were divided into four subpopulations: upper left quadrant - MIE-positive G1 cells (DNA content  = 2n), lower left quadrant - MIE-negative G1 cells, upper right quadrant – MIE-positive S/G2 cells (DNA content >2n), lower right quadrant – MIE-negative S/G2 cells. The relative proportion of each subpopulation is given in percent of total cells. In addition, the percentage of MIE-positive cells in the G1 compartment (both left quadrants) and the S/G2 compartment (right quadrants) is given as a separate information. These latter values are framed in the small boxes at the top corners of each diagram. (B, C) The indicated cell lines were infected with HCMV-AD169 (U373 cells, MOI = 5) or HCMV-TB40/e (HEK293, HeLa, HUVEC, MOI = 1) and harvested at 5 hpi. Cell cycle position was determined by propidium iodide staining (B) or EdU labelling (C), IE expression by IE1/IE2-specific antibody. (B) Flow cytometry data were analyzed as described above. (C) Cells were divided into MIE^+^EdU^−^ (upper left), MIE^−^EdU^−^ (lower left), MIE^+^EdU^+^ (upper right) and MIE^−^EdU^+^ (lower right) fractions. The proportion of each fraction was indicated in percent of total cell number. The small boxes in the top corners of the diagrams display the percentage of MIE-positive cells in the EdU^+^ fractions (left corner) and the EdU^−^ fractions (right corner) respectively.

The block to MIE gene expression in S and G2 phase has been described using the highly attenuated HCMV laboratory strains Towne [11], [12] and AD169 [13]. We first decided to test whether cell cycle dependent IE expression also applies to low-passage strains and clinical isolates, as this would also suggest an *in vivo* relevance for HCMV infection. To this end, we infected human fibroblasts with either high-passage strains AD169, Towne and Davis, low-passage strain Toledo or clinical isolates Merlin, TB40/e and VHL/e and analyzed the cells for MIE gene expression and cell cycle distribution (figure 1A). All strains clearly failed to initiate IE1 and IE2 expression in cells with an S/G2 DNA content. While typically more than 60% of infected G1 cells were IE1/IE2-positive at 3 hpi, only a maximum of 13% S/G2 cells supported MIE expression. Thus, the cell cycle-dependent block to MIE gene expression is a genuine feature of HCMV and not a mere consequence of a reduced virulence attributed to fibroblast adapted laboratory strains.

In contrast to AD169 and Towne, the TB40/e isolate has retained the broad spectrum of cell tropism characteristic for HCMV infections *in vivo*. This enabled us to test whether the S/G2-specific block of MIE expression also accounts to cell types other than fibroblasts. We analyzed primary endothelial cells (HUVEC), two transformed cell lines of epithelial origin (HeLa, HEK293) and the glioblastoma cell line U373-MG (figure 1B and C). All cell types tested were permissive for MIE gene expression in G1 but not S or G2 and hence behaved like primary fibroblasts in this respect. Together these results underscore the general relevance of the cell cycle state for virus-host interaction and in addition, allow more flexibility in choosing informative cellular systems for further specific experimental approaches.

### Prolonged inhibition of DNA replication makes S/G2 cells permissive for MIE gene expression

**Figure 2:**
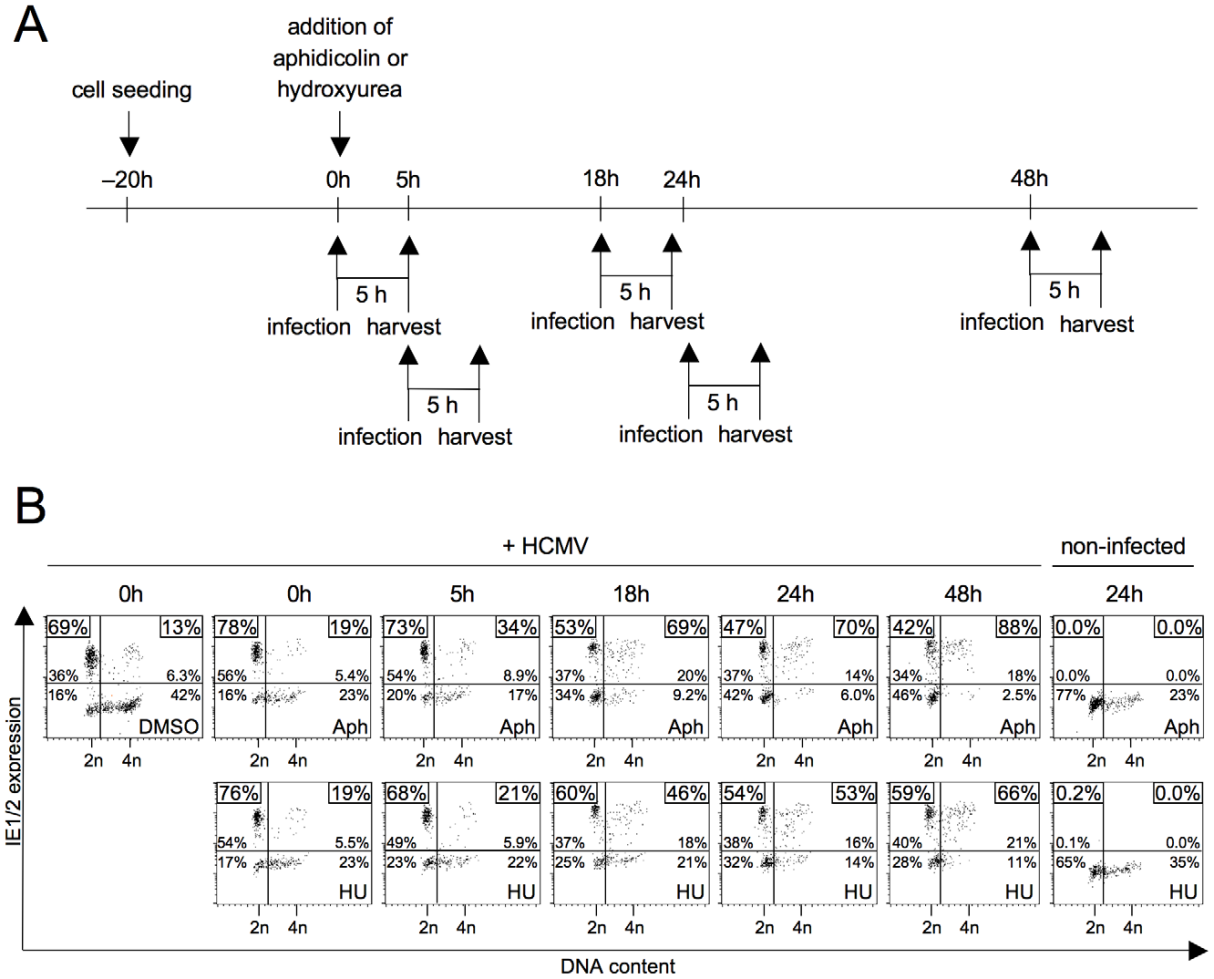
Inhibition of cellular DNA synthesis has no immediate but a delayed effect on the start of MIE gene expression in S/G2. (A) Experimental setup. Proliferating HEL fibroblasts were treated with aphidicolin (Aph) or hydroxyurea (HU) and infected with HCMV-AD169 at the indicated times after drug addition (0 h). At 5 hpi cells were harvested and subjected to propidium iodide and IE1/IE2-specific antibody staining, followed by flow cytometry. (B) Shown are dot plots where the numbers indicate the relative proportion of each quadrant in percent of total cells and (in the small boxes) the percentage of MIE-positive cells within the G1 and S/G2 fractions only, as described in the legend to figure 1.

To identify molecular determinants of the cell cycle-dependent block to HCMV gene expression, we first turned to examine a possible role of cellular DNA synthesis. The rational being that DNA synthesis is the hallmark of S phase in its own right and the observation that the block to IE gene expression was often found more pronounced in S rather than G2 (see figure 1A). Inhibition of DNA replication was achieved by aphidicolin or hydroxyurea treatment. When applying these inibitors together with or a few hours prior to infection we only saw a slight increase of MIE-positive cells in S/G2 compared to DMSO-treated control cells (figure 2) suggesting that ongoing DNA replication is not a cause per se for the inhibition of viral gene expression. However, preincubating cells with the inhibitor for 18 h or longer prior to the 5 h infection period, raised the percentage of MIE-positive cells in the S/G2 compartment to at least the same high level as in G1 cells. This indicates that prolonged inhibition of cellular DNA replication induces secondary events that are able to effectively overcome the S/G2-specific block to HCMV gene expression.

### The DNA damage response relieves the block of MIE gene expression in S/G2

**Figure 3:**
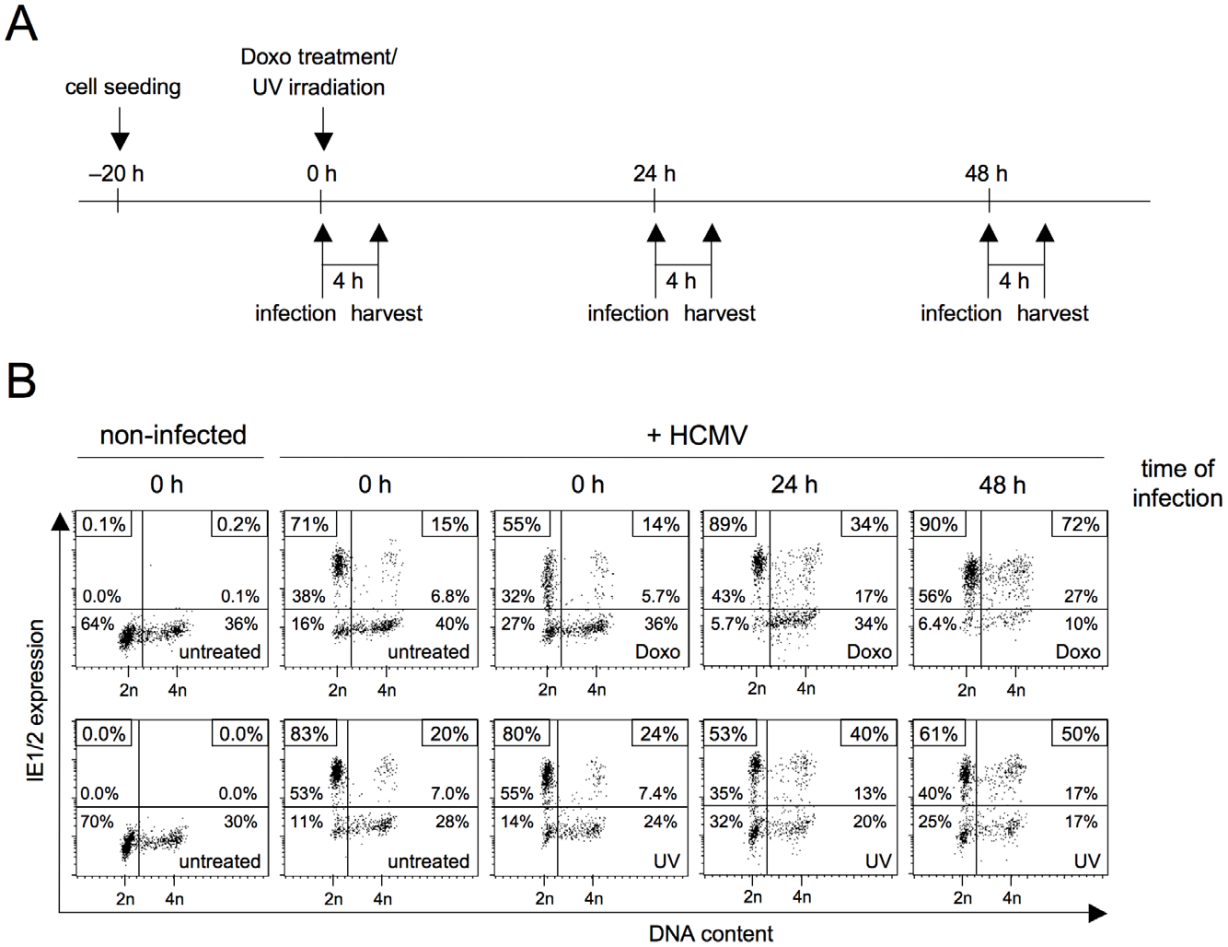
The long-term cellular response to genotoxic stress makes S/G2 cells permissive for MIE gene expression. (A) Experimental setup. Proliferating HEL fibroblasts were treated with sublethal doses of doxorubicin or UV-C radiation. At the indicated times after treatment, cells were infected with HCMV-AD169 and harvested 4 h later. (B) Flow cytometry analysis of DNA content and MIE gene expression, as described in the legend to figure 1.

Chemicals that disrupt replication, such as aphidicolin and hydroxyurea, are known to elicit an ATR-dependent DNA damage response [14], [15]. Thus, the positive influence of replication blockage on MIE gene expression in S/G2 could be a consequence of checkpoint activation rather than the absence of DNA synthesis per se. To address this point, we directly induced DNA damage and chose two independent ways to do so, namely doxorubicin treatment and ultraviolet (UV) irradiation. To cover both early and delayed effects of checkpoint activation on viral gene expression, cells were infected at different time points after induction of DNA damage (figure 3A). Cells that were infected immediately after DNA damage showed the same pattern of cell cycle-dependent MIE gene expression as untreated cells (figure 3B). In contrast, at 24 h and especially at 48 h after DNA damage many cells with an S/G2 DNA content had become permissive for MIE gene expression. This finding demonstrates two things. First, induction of the DNA damage checkpoint is indeed effective in creating a supportive environment for viral MIE gene expression in S/G2. Second, the events leading to increased permissiveness are likely to be part of the long-term and not the fast but transient response to cellular DNA damage.

### Checkpoint-induced rescue of MIE gene expression allows the HCMV lytic cycle to proceed with normal kinetics

**Figure 4:**
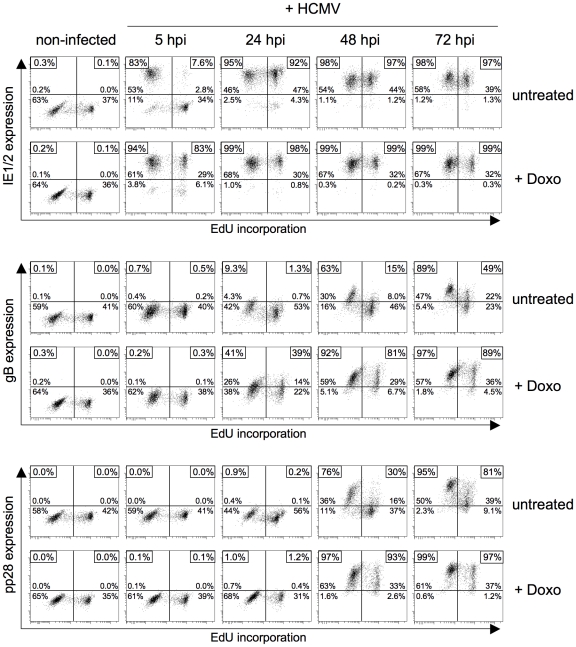
Infection at late times after genotoxic stress allows the HCMV replicative cycle to proceed in S/G2 cells with similar kinetics as in normal G1 cells. Proliferating HEL fibroblasts were incubated for 60 min with EdU to label S phase cells. After removal of EdU, cells were treated with doxorubicin (+doxo) or left untreated. 24 h post doxorubicin treatment, or – in the case of untreated cells – immediately after EdU labelling, cells were infected with HCMV-AD169. Cells were harvested at the indicated times post infection and analyzed for EdU incorporation and HCMV MIE (IE1/2), early (gB) and late (pp28) gene expression by flow cytometry. The percentage of cells staining positive for the different viral gene products was displayed as described in the legend to figure 1C.

The non-permissive nature of S/G2 cells causes a long delay in the HCMV replicative cycle, as viral gene expression cannot be initiated before cells have divided and reentered G1 [11], [12]. To investigate whether the DNA damage-induced rescue of MIE gene expression is also sufficient to trigger the consecutive gene expression program of the normal replicative cycle in S/G2 cells, we compared the kinetics of MIE, early and late gene expression in untreated and doxorubicin treated cells (figure 4). Consistent with a previous report [11], after infection of untreated S phase cells the program of lytic gene expression lagged at least 24 h behind that of infected G1 cells. In contrast, doxorubicin treated S phase cells were able to support not only MIE but also the subsequent steps of the viral gene expression program without delay. Of note, this also includes the pp28 gene. The fact that expression of true late genes like pp28 depends on the onset of viral DNA synthesis indicates that also viral DNA replication was initiated in S/G2 cells with normal kinetics. Taken together, these data clearly suggest that with the DNA damage-dependent release of MIE gene expression the major obstacle to HCMV replication is removed from S phase cells and infection proceeds with the normal kinetics known from infected G0/G1 cells.

### P53 is of central importance to the rescue of MIE gene expression in S/G2

**Figure 5:**
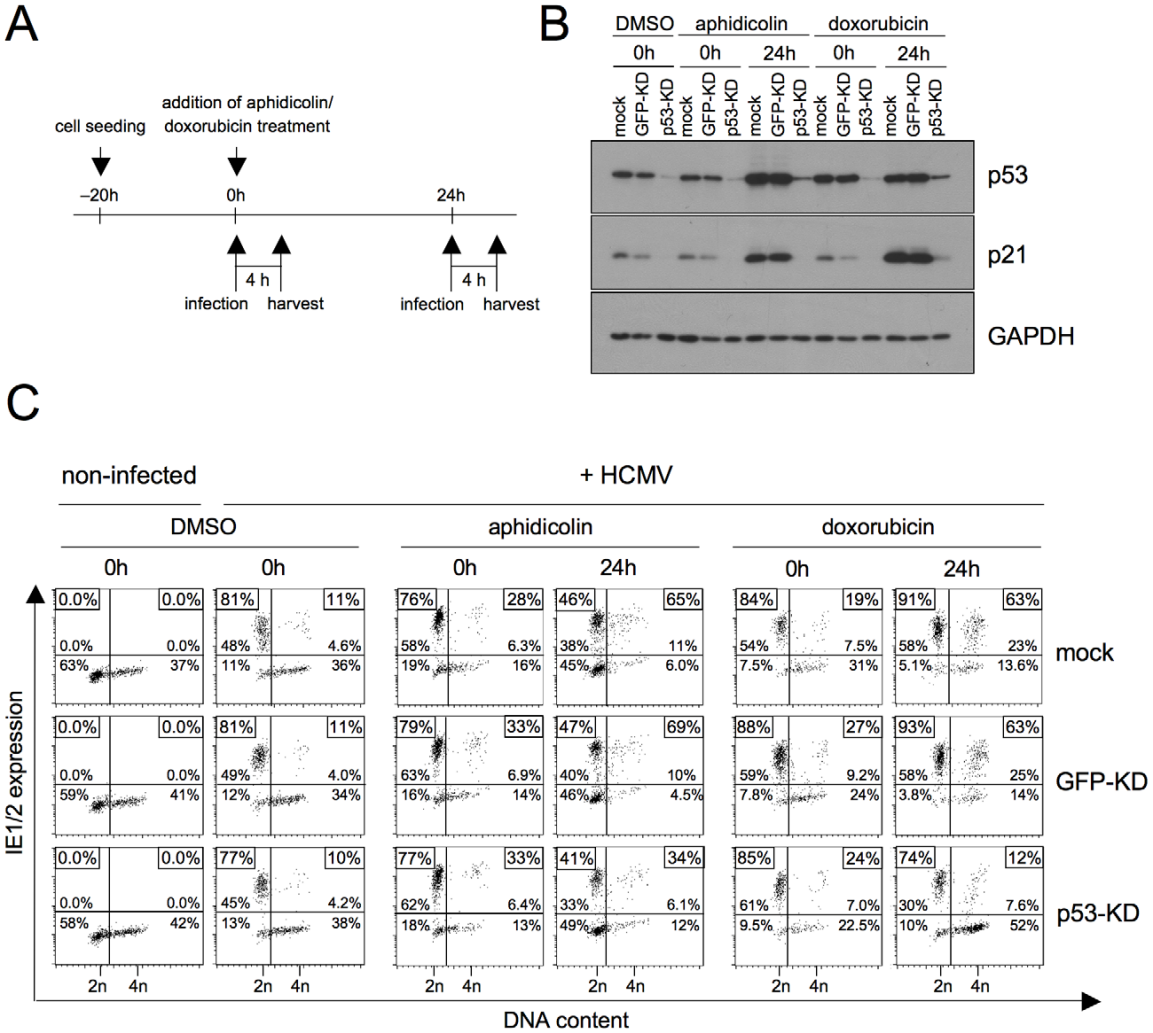
P53 is required for the checkpoint-dependent rescue of MIE gene expression in S/G2. HEL fibroblasts were stably transduced with lentiviruses expressing p53 or GFP-specific shRNAs. The resulting knock-down (KD) cells and a mock-infected control were treated with aphidicolin or doxorubicin as indicated. Immediately after treatment (0 h) or 24 h later cells were infected with HCMV-AD169 (A). Cells were harvested 4 h post infection and analyzed for expression of p53, p21 and GAPDH by immunoblotting (B) and for MIE expression versus DNA content by flow cytometry (C).

The p53 tumor suppressor protein governs the long-term cellular response to genotoxic stress by inducing a p21-dependent cell cycle arrest or apoptosis [16]. Since the observed rescue of viral gene expression in S/G2 cells after DNA damage follows long-term kinetics, we next asked whether this rescue depends on p53-dependent signalling. To this end we made use of HEL fibroblasts with a stable p53 knockdown [17]. Using the same experimental setup as before, we treated p53 knockdown (p53-KD) and appropriate control (mock-transduced and GFP-KD) fibroblasts with aphidicolin or doxorubicin and infected them immediately (0 h) or 24 h after treatment (figure 5A). Immunoblot analysis confirmed that p53-KD but not control cells resist the aphidicolin and doxorubicin-induced upregulation of p53 and p21 (figure 5B). The increase of p53 expression in control cells between 0 and 24 h was accompanied by the expected increase in permissiveness for MIE gene expression in S/G2 (figure 5C). In contrast, p53-KD cells failed to upregulate MIE genes in S/G2 cells rather showing the typical G1-restricted pattern of MIE gene expression and this was regardless of whether infection was initiated at early or late times after DNA damage or inhibition of DNA replication. This clearly demonstrates that the rescue of HCMV gene expression in S/G2 relies on p53-dependent checkpoint signalling both, after induction by genotoxic (doxorubicin) or replicative (aphidicolin) stress.

Different to our approach, a study by Fortunato et al. employed 24 h aphidicolin treatment to synchronize cells at the G1/S-transition before HCMV infection [12]. At the time of infection they released the cells from the aphidicolin block, enabling cells to recover and cycle through S/G2/M/G1. As the continuous presence of the drug is needed to keep the replication checkpoint and p53-dependent signalling active, it is not contradictory to our findings that their synchronization protocol had no major effect on the percentage of MIE-positive cells in S/G2.

**Figure 6:**
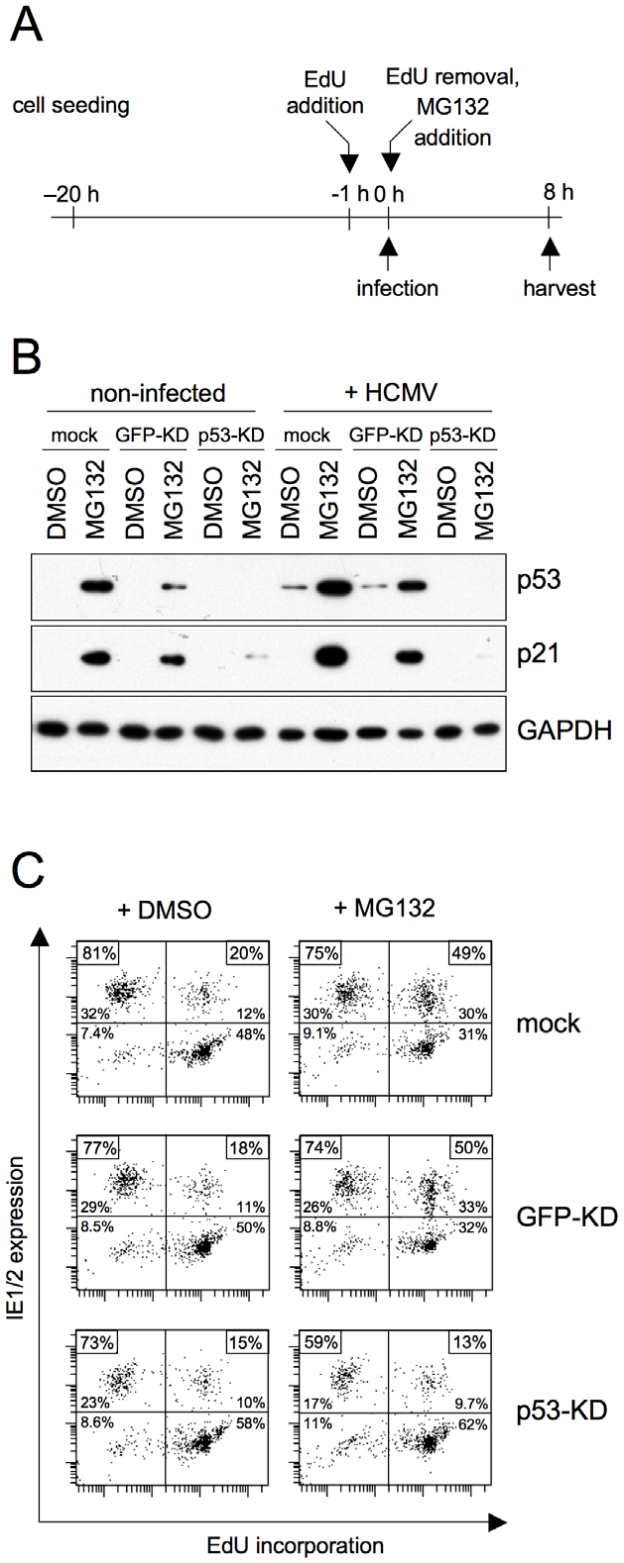
The positive effect of proteasome inhibition on MIE gene expression in S/G2 is mediated by p53 stabilization. (A) Experimental setup. Proliferating HEL fibroblasts and the indicated knock-down derivatives were first incubated with EdU to label S-phase cells. After removal of EdU, cells were infected with HCMV-AD169 or left uninfected. Together with the virus, the proteasome inhibitor MG132 was added to the cells and was not removed before harvest at 8 hpi. A solvent control (DMSO) was included. (B) Cells were examined for expression of the indicated proteins by immunoblot analysis. (C) Flow cytometry analysis of MIE gene expression and EdU incorporation. The percentage of MIE-positive cells in the EdU-positive and EdU-negative fractions respectively were displayed as described in the legend to figure 1C.

P53 is a short-lived protein whose abundance is controlled by Mdm2-mediated ubiquitination and subsequent degradation by the 26S proteasome [18]. It has been known for some time that the block to MIE gene expression in S phase can be overcome by proteasomal inhibitors [12]. However, the reason for that remained elusive. Given the above results we rationalized that with respect to MIE expression in S phase p53 might be the limiting target of proteasomal inhibition. Like DNA damage proteasomal inhibition leads to p53 stabilisation and consequently to checkpoint activation. To directly address this hypothesis, we infected p53-KD and control fibroblasts in the presence of the proteasome inhibitor MG132 (figure 6A). As expected, after 8 h of MG132 treatment both p53 and its target p21 had strongly accumulated in non-infected and HCMV-infected control cells and this effect was largely suppressed in p53-KD cells (figure 6B). The following analysis of cell cycle dependent viral MIE gene expression revealed that the p53 status was indeed crucial for the increased permissivenes of S phase cells after proteasome inhibition. In the presence of p53, MG132 treatment resulted in a marked increase of MIE-positive S phase cells from 20% to 50% (figure 6C). In contrast, in p53-negative cells proteasomal inhibition had no influence on MIE gene expression that even dropped from 15% to 13% in the S phase compartment. Thus, the finding that unrelated agents such as aphidicolin, doxorubicin and MG132 rescue MIE gene expression in S phase fibroblasts is a consequence of their shared ability to induce the p53 tumor suppressor protein.

### The effect of p53 upregulation on MIE gene expression is mediated by p21

**Figure 7:**
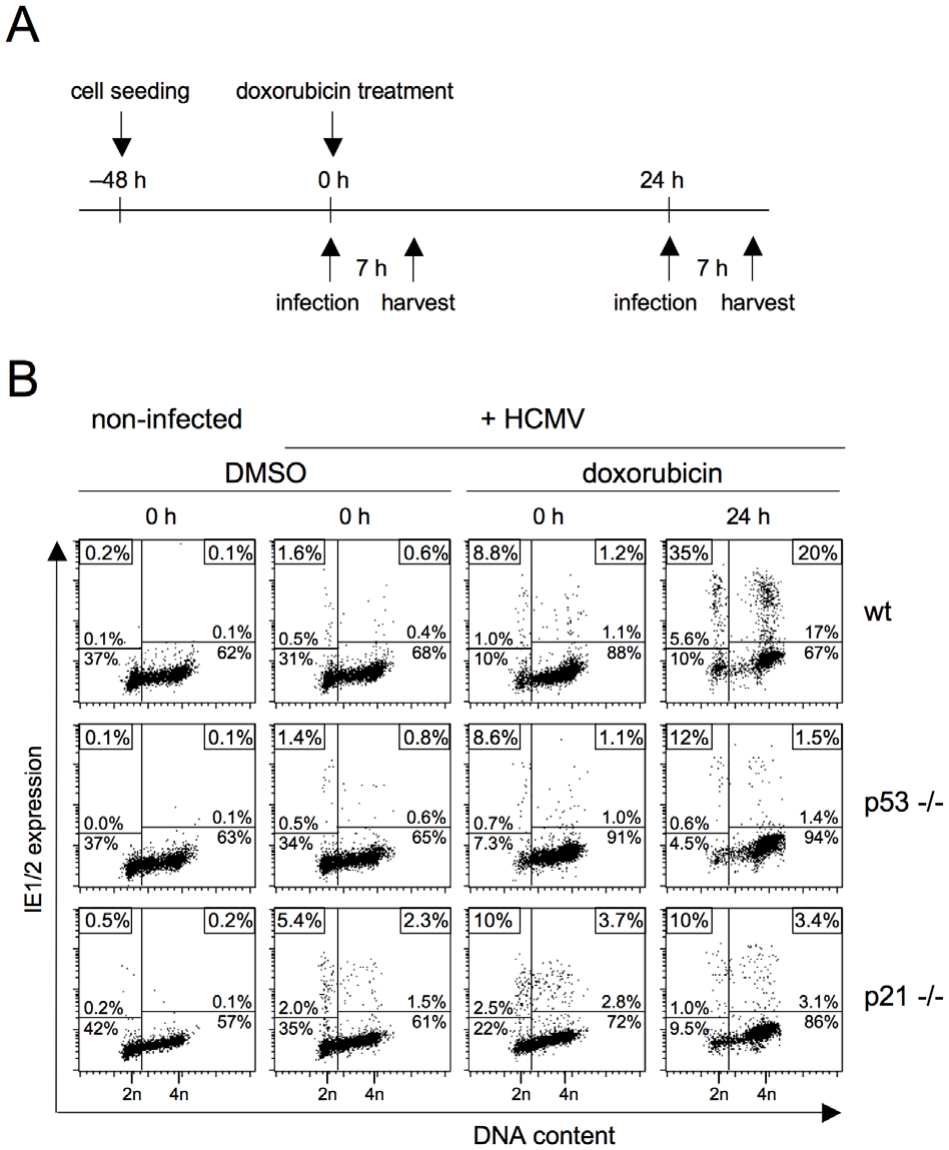
DNA damage-dependent rescue of MIE gene expression in S/G2 depends on the CDK inhibitor p21. Proliferating HCT116 wild type (wt), p53−/− and p21−/− cells were treated with doxorubicin and infected thereafter with HCMV-TB40/e at an MOI of 1. Infection was carried at 0 or 24 h post doxorubicin treatment (A). Cells were harvested at 7 hpi and analyzed for DNA content and MIE gene expression using flow cytometry (B), as described in the legend to figure 1.

One of the main effectors of p53-dependent signalling is the CDK inhibitor p21 [19]. To directly test if p21 is required for the p53-dependent release of MIE gene expression in S/G2 we focussed on the HCT116 cell system where somatic knockouts of both p53 and p21 are available [20]. Because HCT116 cells are of epithelial origin and therefore non-permissive for fibroblast adapted HCMV laboratory strains [21] we used the endotheliotropic TB40/e isolate for infection. As before, we applied doxorubicin to induce via DNA damage the p53-p21 checkpoint axis and analyzed MIE gene expression after infection at early (0 h) and late times (24 h) post doxorubicin treatment (figure 7A). It appeared that untreated HCT116 cells in G1 as well as in S/G2 only weakly support MIE gene expression (see left part of figure 7B). For wild type (wt) and p53−/− cells, the proportion of MIE-positive cells was below 2% in all cell cycle phases, only p21−/− cells appeared to be a slightly more supportive of viral gene expression with up to 5.4% MIE-positive cells in G1. However, after exposure to doxorubicin the permissiveness of HCT116 cells increased remarkably (see right hand panels of figure 7B). A first very minor increase was already visible at 0 h, yet proved to be independent of p53 and p21. After 24 h, the number of permissive wt cells was increased to 35% in G1 and 20% in S/G2. This late effect of doxorubicin treatment was almost completely prevented in p53 and p21 knockout cells.There, compared to wt cells, the percentage of MIE-positive cells was 3-fold (p53−/−) and 3.5-fold (p21−/−) lower in G1 and even 13-fold (p53−/−) and 6-fold (p21−/−) lower in S/G2. First, these results show that in the case of HCT116 cells, viral gene expression in G1 seems to be subject to the same repressive mechanism as in S/G2. Second, in addition to p53 the rescue of MIE gene expression also and equally depends on p21, suggesting that p21 represents the critical effector of p53 in this context.

### CDK inhibition by roscovitine relieves the block to the initiation of MIE gene expression during S/G2 in a direct, p53 independent manner

**Figure 8:**
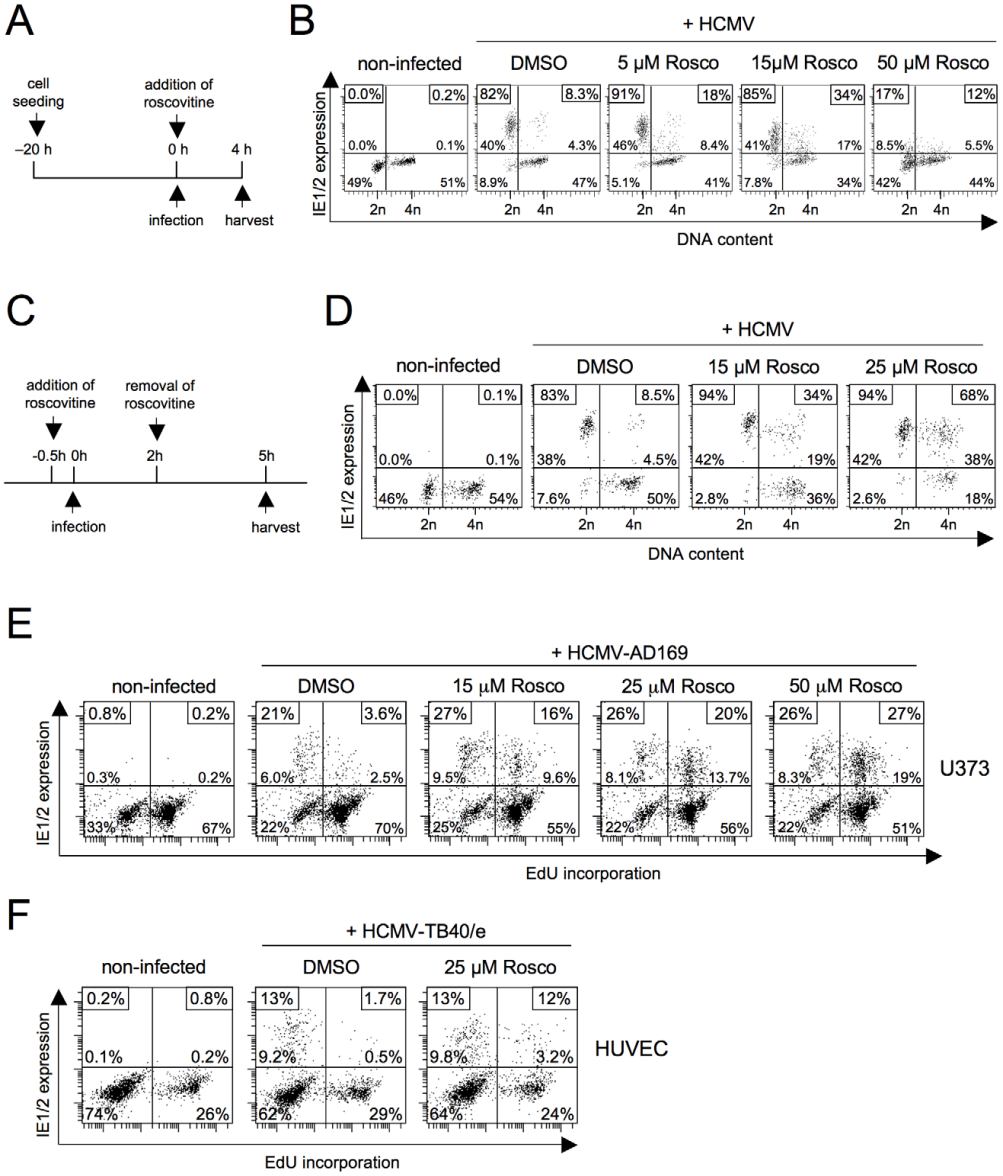
Roscovitine treatment efficiently abrogates the block of MIE gene expression in S/G2. (A) Experimental setup of B. Proliferating HEL fibroblasts were infected with HCMV-AD169. Roscovitine, at the indicated concentrations, was added to the cells at the time of infection. It was left on the cells until harvest at 4 hpi. (C) Experimental setup of D. This time, roscovitine treatment was started 30 min before the fibroblasts were infected with HCMV. Treatment was stopped at 2 hpi and infected cells were maintained for further 3 h in regular growth medium. (B, D) MIE gene expression and DNA content of roscovitine treated cells were analyzed by flow cytometry. DMSO treated cells were also analyzed to control for solvent effects. (E, F) Proliferating U373 and HUVEC cells were incubated with EdU for 60 min to label cells undergoing DNA synthesis. After removal of EdU, cells were infected with HCMV and harvested 5 h later. Where indicated, roscovitine was transiently added to the cells according to the schedule described in C. After harvest, cells were analyzed for EdU incorporation and IE1/2-expression by flow cytometry, as detailed in the legend to figure 1.

The best understood checkpoint effector functions of p21 are the inhibition of DNA replication by PCNA binding and the inhibition of CDK2 and CDK1 activity [19]. To test whether the p21-dependent effect on MIE gene expression relies on CDK inhibition, we infected fibroblasts in the presence of the pharmacological CDK inhibitor roscovitine (also known as CYC202 or seliciclib). In a first set of experiments, roscovitine was applied in parallel with viral infection, i. e. left on the cells from the beginning of infection until cell harvest at 4 hpi (figure 8A). The effects we observed were clearly dose-dependent. Low doses (5 µM) of roscovitine resulted in a modest increase from 8% to 18% MIE-positive cells in S/G2 (figure 8B) and at medium concentrations (15 µM) the number further increased to 34%. However, at the same time and independent of the cell cycle position the average MIE expression level per cell dropped significantly with increasing concentrations of roscovitine such that high doses (50 µM) finally led to an almost complete loss of MIE gene expression in all cell cycle phases. Thus, roscovitine exerts two opposing effects during the first hours of HCMV infection. The cell cycle independent, negative effect on MIE gene expression has been described [22] and attributed to inefficient viral transcription caused by the roscovitine-mediated inhibition of CDK7 and CDK9 [23], [24]. However, more pertinent to the question examined here and in good agreement with the above data there is a clear positive effect of roscovitine on MIE gene expression. In a previous study roscovitine (15 µM) was left on HCMV-infected cells until harvest at 12 hpi and did not increase the percentage of IE-positive cells in S/G2 [12]. In this setting, the negative influence of CDK inhibition on ongoing viral gene expression probably outweighed the positive effect on its initation.

In order to better discriminate between the two opposing activities of CDK inhibition, we next modified our experimental setup. To this end, we only allowed roscovitine on infected cells for two hours before harvesting cells at 5 hpi (figure 8C). Thus, roscovitine should have exerted its positive effect on the initiation of MIE without negatively affecting further viral MIE protein accumulation. Indeed, this setting avoided the negative impact of CDK inhibition resulting in a further doubling of MIE-positive cells in S/G2 (from 34% to 68%) when increasing the roscovitine concentration from 15 to 25 µM (figure 8D). This further supports the view that CDK activity acts as a strong inhibitor of the initiation of viral MIE gene expression during the onset of lytic infection. Roscovitine treatment also rescued S/G2 phase-specific MIE gene expression in U373 and HUVEC cells (figure 8E). Even at high concentrations of roscovitine (50 µM in the case of U373) MIE gene expression was not impaired to any extent in these cells reaching high and comparable levels in the S/G2 and G1 cell cycle phases.

**Figure 9:**
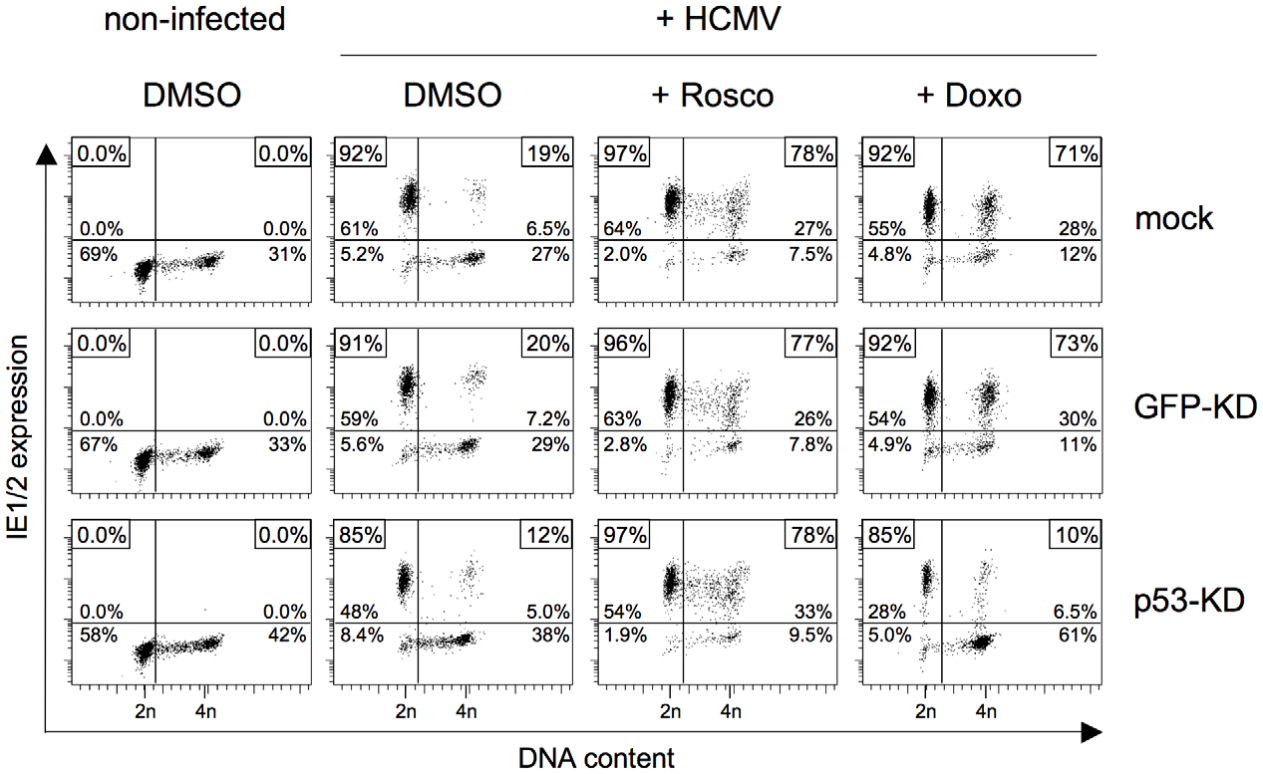
The roscovitine-mediated rescue of MIE gene expression in S/G2 cells is p53 independent. The indicated knock-down fibroblasts were treated with doxorubicin (+ Doxo) and infected by HCMV 24 later. Or cells were treated with 25 µM roscovitine (+ Rosco) and infected with HCMV shortly after the start of treatment, as detailed in figure 8C. Cells were harvested at 5 hpi and analyzed for MIE expression and DNA content, as described in the legend to figure 1.

It has been shown that, in addition to CDK inhibition, roscovitine treatment can also trigger p53-dependent signalling [25], [26]. Although the observed kinetics made it unlikely that the effect of roscovitine was mediated via p53 signalling, we aimed at formally excluding this possibility to further unravel the importance of a direct inhibitory CDK function on initiation of HCMV MIE gene expression. To this end we analyzed whether roscovitine – unlike doxorubicin, aphidicolin or MG132 (see above) – is still effective in the absence of p53. Using p53 positive control fibroblasts, the direct comparison to doxorubicin treatment revealed that short-term treatment with roscovitine was as efficient in creating a permissive state in S/G2 cells as the long-term response to DNA damage. However, while the effect of doxorubicin was completely abolished in p53-KD cells, the strong effect of roscovitine proved to be p53 independent (figure 9).

In summary, these data demonstrate that short-term treatment with roscovitine can overcome the block to HCMV MIE gene expression in S/G2. This roscovitine-mediated rescue resembles the p53 and p21 dependent MIE expression after long-term checkpoint activation but acts downstream from and independent of p53, directly targeting CDK activity. Thus, CDK activity exerts a strong negative effect at the initiation state of HCMV lytic infection.

### CDK inhibition prevents silencing of MIE gene expression in the NT2 latency model

Latent infection arguably represents the most relevant *in vivo* situation where MIE gene expression of HCMV is dominantly repressed. To address the important question as to whether CDK activity could also contribute to maintain the block to IE expression in latently infected cells, we made use of the embryonic carcinoma cell line NT2, a well-accepted *in vitro* model for HCMV latency [27]. In undifferentiated, pluripotent NT2 cells MIE gene expression is blocked and HCMV establishes a quiescent infection. The block can be relieved by chemical induction of neuronal differentiation [28], [29], [30], [31]. This resembles the differentiation state-dependent permissiveness of cells of the myeloid lineage, a major site of HCMV latency *in vivo* [32].

**Figure 10:**
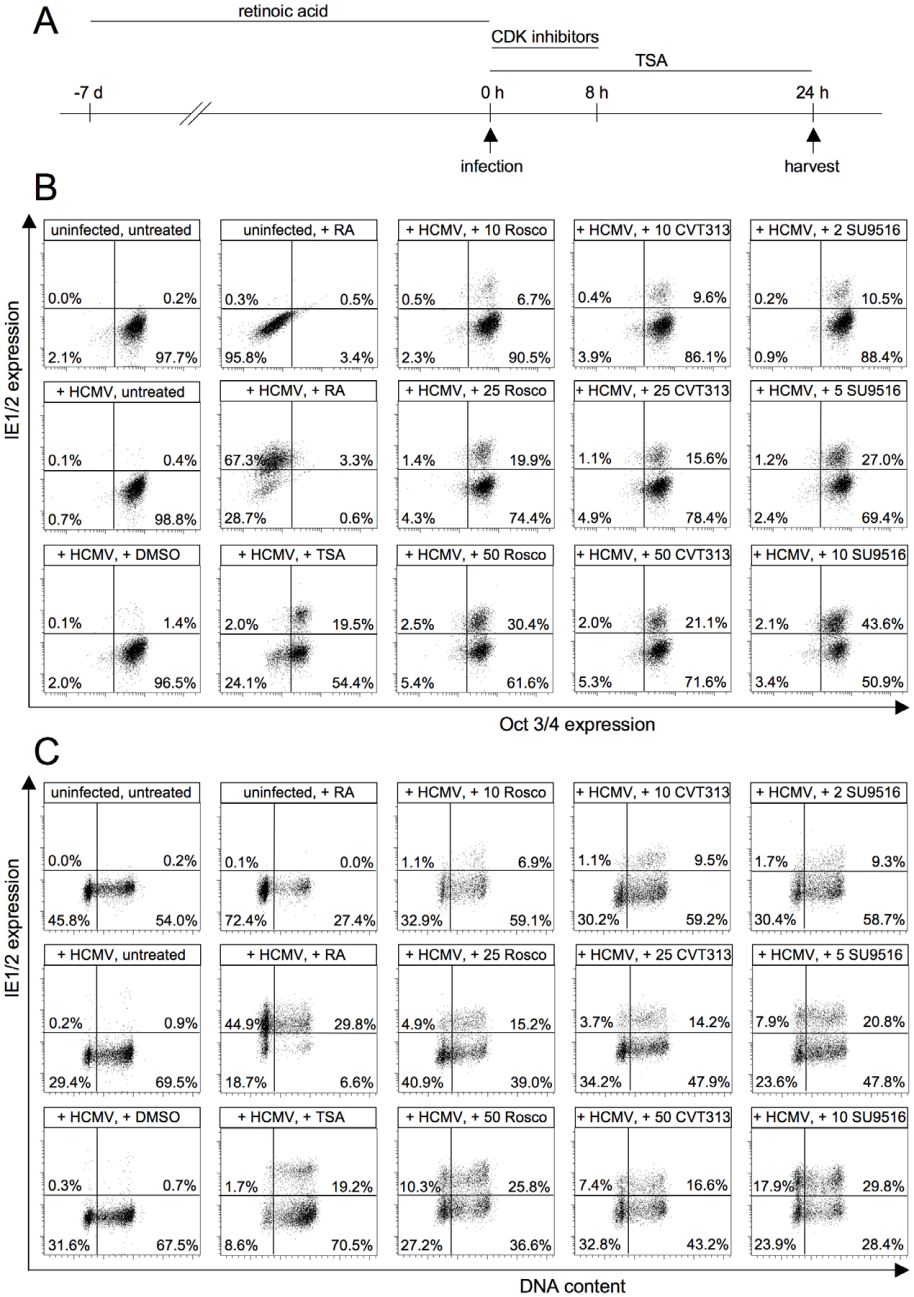
CDK inhibition relieves the block of MIE gene expression in undifferentiated NT2 cells. (A) Experimental setup. NT2 cells were infected with HCMV-TB40/e (MOI = 5) in the presence of the cyclin-dependent kinase (CDK) inhibitors roscovitine (Rosco), CVT313, SU9515, the histone deacetylase inhibitor trichostatine A (TSA) or solvent (DMSO). CDK inhibitors were used at concentrations from 10 to 50 µM (Rosco, CVT313) and from 2 to 10 µM (SU9516) as indicated. The different compounds were left on the cells for the indicated lengths of time. In addition, NT2 cells were infected after a 7-day exposure to retinoic acid (RA). All cells were harvested at 24 hpi. (B) Cells were stained for IE1/2 and Oct3/4 expression and analyzed by flow cytometry. Shown are dot plots where cells were divided into four subpopulations: MIE^−^Oct^−^ (lower left quadrant), MIE^+^Oct^−^ (upper left quadrant), MIE^+^Oct^+^ (upper right quadrant), MIE^−^Oct^+^ (lower right quadrant). The proportion of each subpopulation is given as percent of total cells. (C) Cells were stained for IE1/2 expression and DNA content and analyzed by flow cytometry. The depicted cells were divided into the following subpopulations: MIE^−^G1 (lower left quadrant), MIE^+^G1 (upper left quadrant), MIE^+^S/G2/M (upper right quadrant) and MIE^-^S/G2/M (lower right quadrant) cells. The proportion of each subpopulation is given in percent of total cells.

To analyze a possible function of CDKs for the permissiveness of HCMV in NT2 cells, we adjusted the experimental set up to the slower kinetics of viral gene expression in NT2 cells (figure 10A). Two positive controls were included in the experiment. Retinoic acid (RA)-induced differentiation (as indicated by the downregulation of the pluripotency marker Oct4) enabled MIE gene expression in 70% of all cells (figure 10B). Trichostatin A (TSA)-mediated inhibition of histone deacetylases led to 21% IE1/2-positive cells. However, in this case the rescue of MIE gene expression was differentiation-independent, which is consistent with previous reports [33], [34]. Intriguingly, transient CDK inhibition also increased the permissiveness of NT2 cells for MIE gene expression. This was shown for three different CDK inhibitors. Besides roscovitine we used another 2,6,9-substituted purine analogue (CVT313) and a structurally unrelated compound (SU9516). SU9516 proved to be most effective leading to up to 44% IE1/2-positive cells after 24 h of infection compared to only 1.4% in the DMSO-treated control. Of note, this induction was not a consequence of CDK inhibitor-induced differentiation since IE-expressing NT2 cells remained undifferentiated by means of undisturbed Oct3/4 expression. This result suggests that the inhibitory function of CDKs on HCMV lytic gene expression is not restricted to the cell cycle but is likely to have broader relevance for states of inhibited IE expression. In line with this reasoning, we were able to show that the induction of MIE gene expression by transient CDK inhibiton can occur in all cell cycle phases of NT2 cells even though S/G2 cells reacted most sensitive at low, suboptimal concentrations of inhibitors (figure 10C).

**Figure 11:**
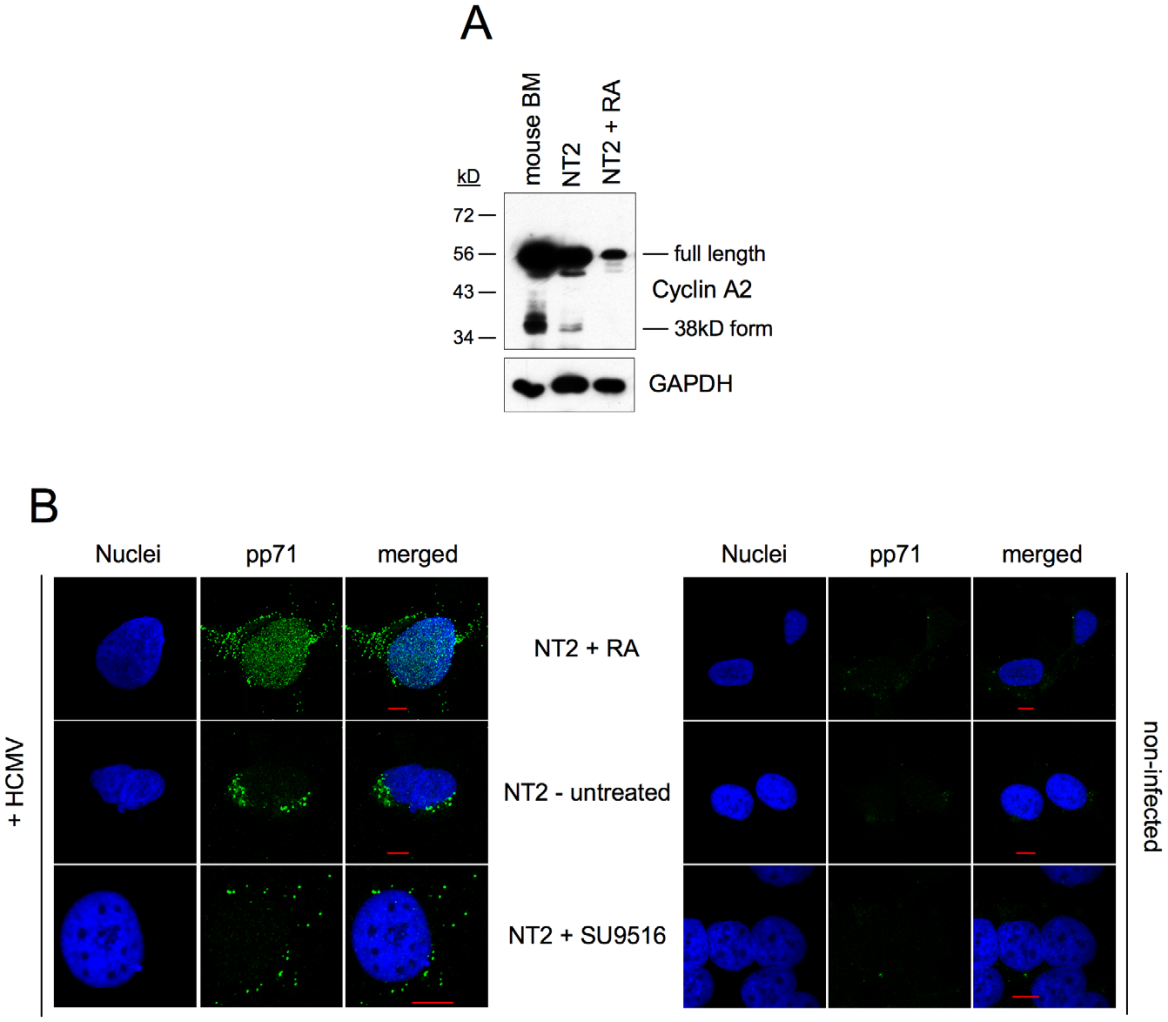
Induction of MIE gene expression by CDK inhibition does not require nuclear localization of pp71. (A) Undifferentiated NT2 cells, differentiated NT2 cells (treated with RA for 7 days) and mouse bone marrow (BM) cells were analyzed for Cyclin A2 expression by immunoblot analysis. GAPDH expression was analyzed to control for equal loading. (B) Undifferentiated and differentiated NT2 cells were infected with HCMV-TB40/e (MOI = 5). Where indicated, SU9516 at 10 µM final concentration was added together with the virus. At 6 hpi cells were analyzed for subcellular localization of pp71 (green) by immunofluorescence microscopy. Nuclei were stained with DAPI fluorochrome (blue). Non-infected cells (right panel) were analyzed to control for background staining. Scale bars (red)  = 10 µm.

Given the cell cycle-independent nature of MIE induction by CDK inhibitors, we asked whether the permissiveness of NT2 cells might correlate with the expression of a constitutively active 38kD-form of Cyclin A2 that was recently described in mouse bone marrow and human myeloid precursor cells [35], [36]. Indeed this form was detectable in undifferentiated but not differentiated NT2 cells (figure 11A). In addition, the expression of full-length Cyclin A2 dropped significantly during retinoic acid-induced differentiation. This is consistent with published data [37] and might at least partially explain the observation that differentiated NT2 cells express MIE genes in a completely cell cycle-independent manner (figure 10C).

An already described determinant of MIE expression in NT2 cells is the differentiation-dependent nuclear localization of the viral tegument protein pp71 [38]. Nuclear localization of pp71 facilitates MIE gene expression by neutralizing the Daxx-mediated cellular intrinsic immune defense [39]. To address the question as to whether the rescue of MIE expression by CDK inhibition works via nuclear translocation of pp71 we compared the subcellular localization of pp71 under conditions of CDK inhibition and retinoic acid-induced differentiation (figure 11B). We found that the cytoplasmic localization of pp71 in untreated cells was not affected upon CDK inhibition. In contrast, retinoic acid-treated cells contained a large fraction of nuclear pp71 as expected. This suggests that CDK inhibition and pp71 nuclear translocation trigger independent mechanisms to induce MIE gene expression. Considering that after retinoic acid treatment MIE expression occurs in a cell cycle independent fashion (figure 10C) and pp71 is able to enter the nucleus (figure 11B and [38]) it appears that both mechanisms can respond to differentiation and therefore might act synergistically.

## Discussion

**Figure 12:**
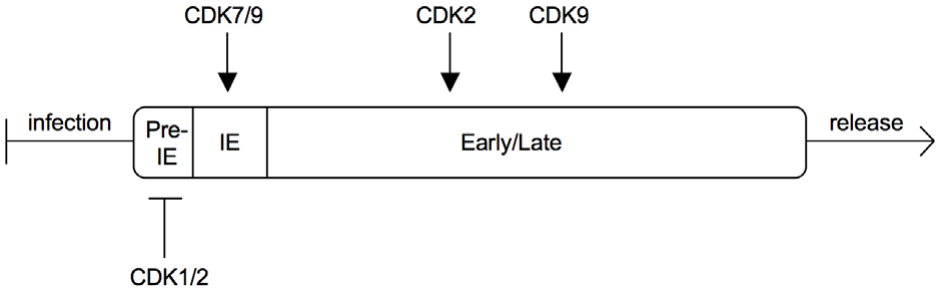
Sequential regulation of the HCMV replication cycle by different CDKs. After virus entry a cell cycle-regulated CDK is able to prevent the onset of IE gene expression. Once IE expression has started, CDK7 and 9 become an essential part of the viral transcription machinery. At early and late times of infection CDKs are needed for proper function and localization of viral substrates.

Here we show for the first time that CDK activity negatively controls the onset of HCMV gene expression. This finding was unexpected because HCMV, like many other viruses, was previously shown to be subject of positive regulation by CDKs. Apparently, the effect of CDK activity on HCMV varies depending on the phase of infection and the type of CDK (see figure 12). At a pre-immediate-early stage of infection, an S/G2-specific, probably Cyclin A2-dependent (see below) CDK prevents the initiation of IE gene expression at the level of transcription (this study and our own unpublished data). Once IE expression has been initiated, CDK activity is required for accurate processing and accumulation of viral transcripts [22]. This correlates with recruitment of CDK7 and CDK9 to the sites of viral transcription where they catalyze hyper-phosphorylation of the RNA polymerase II C-terminal domain [23], [24]. At later times CDK activity is needed for proper expression, modification and localization of pUL69, pUL83 and other HCMV proteins and for efficient production of viral progeny [40], [41], [42]. Both CDK2 [43] and CDK9 [40] but not CDK1 [44] are likely to contribute to these late effects. When CDKs are inhibited througout infection, the net effect is an almost complete suppression of HCMV replication [43], [44]. Accordingly, CDKs have been suggested as targets for anti-HCMV therapy [45], [46]. However, our data raise a possible caveat about the use of CDK inhibitors as antiviral drugs because they might favour HCMV induction in non-permissive cell types and during latent or latent-like infections.

Several lines of evidence argue that the CDK activity leading to inhibition of MIE gene expression in S/G2 is provided by Cyclin A2-CDK1/2. First, CDK1 and CDK2 are the only CDKs inhibited by both p21 (binds CDK1, 2, 4 and 6 [47]) and roscovitine (targets CDK1, 2, 5, 7 and 9 [48]). Second, Cyclin A2-dependent kinase activity is induced at the G1/S transition and remains high until early prometaphase [49], thus constituting the only Cyclin-CDK activity profile that matches the non-permissive cell cycle window for HCMV. Third, HCMV induces high levels of Cyclin E and B-dependent kinase activity but represses Cyclin A2 in an IE2-dependent manner [11], [50], [51], [52]. This points towards a model whereby HCMV – as soon as MIE gene expression has started – evades the inhibitory influence of Cyclin A2-CDK activity. Also in the case of latent-like infection the Cyclin A2-CDK status appears to perfectly correlate with the ability of cells to support MIE gene expression. Retinoic acid-induced differentiation which prevents the establishment of MIE quiescence in NT2 cells is also known to cause downregulation of Cyclin A2 (see also figure 11A), induction of the CDK inhibitor p27 and inhibition of CDK2 activity [37]. Work is ongoing in our laboratory to determine the role of Cyclin A2-dependent kinase activity during lytic infection as well in experimental models of HCMV latency.

To understand the mechanism of CDK dependent inhibition of HCMV MIE gene expression it will be essential to know the CDK substrate mediating this control. One possibility is that a viral regulator of MIE gene expression changes its activity, expression or localization depending on the CDK status of the host cell. Examples for such viral sensors of cellular CDK activity are the bovine papillomavirus (BPV) protein E1 and the apoptin protein of chicken anemia virus. BPV-E1, an essential factor for the initiation of viral replication, is inactivated and marked for nuclear export in S/G2 by Cyclin A2-CDK2-dependent phosphorylation [53]. In contrast, apoptin, a tumor selective inducer of apoptosis, requires phosphorylation by Cyclin A2-CDK2 for nuclear localization and cell death induction [54]. In the case of HCMV, the CDK substrate needs to be present at pre-IE times of infection, so the best viral candidate factors would be tegument proteins which are delivered as part of the HCMV virion to the host cell. Importantly, a number of tegument proteins have already been decribed to exert functions prior to MIE gene expression [55] and therefore represent possible targets for such a claimed CDK-dependent mechanism. Candidates include pUS24 [56], pUL26 [57], [58], pUL28/29 [59], pUL35 [60], [61], pUL47 [62], pUL76 [63]. The differentiation-dependent subcellular localization of pp71 that regulates its Daxx-neutralizing function in NT2 and THP-1 cells [38], [39] already provides a proof of principle for the control of HCMV tegument proteins by cellular factors immediately after infection. Moreover, the example of the herpes simplex virus protein VP16 demonstrates that the availability of a tegument protein can be decisive for the cell cycle sensitivity of herpesviral IE gene expression [64].

How might HCMV benefit from a CDK-sensitive mechanism controlling MIE gene expression? In a simple view, it enables the virus to synchronize the onset of its lytic cycle with G0/G1 - the cell cycle phase that is considered to be most supportive for virus replication. An alternative scenario is that CDK activity is a downstream constituent of a p53 sensitive switch that can operate between latent and lytic infection. In this case cell cycle dependency of HCMV may be an additional by-product. P53 is a coordinator of cellular responses to different kinds of stress including inflammatory [65] and oncogenic stress [66]. Given that HCMV is frequently found reactivated in inflammatory diseases and cancer [67], the possibility that activated p53 triggers lytic gene expression via CDK inhibition appears an attractive option.

## Materials and Methods

### Cells

Human embryonic lung (HEL) fibroblasts (Fi301, obtained from the Institute of Virology, Charité, Berlin, Germany) were maintained in Eagle's minimum essential medium (EMEM) supplemented with Earle's balanced salt solution, 25 mM HEPES, 1 mM sodium pyruvate, 2 mM L-alanyl-L-glutamine, nonessential amino acids, 0.75 ‰ (w/v) sodium bicarbonate, 50 µg/ml gentamicin and 10% fetal bovine serum (FBS). Human umbilical vein endothelial cells (HUVEC) were obtained from Lonza (Walkersville, MD, USA) and maintained in EGM medium (Lonza) following the manufacturer's instructions. The generation of stable p53 and GFP knockdown derivatives was described elsewhere [17]. U373-MG, HEK293 and HeLa cells (all from ATCC, Manassas, VA, USA) were maintained in Dulbecco's modified Eagle medium (DMEM) supplemented with 10% FBS, 2 mM L-alanyl-L-glutamine, 100 U/ml penicillin and 100 µg/ml streptomycin. The human colon carcinoma cell line HCT116 and its p53−/− and p21−/− derivatives were obtained from Bert Vogelstein (Baltimore, MD, USA) and maintained in McCoy's 5a medium supplemented with 10% FBS, 5 mM glutamine, 100 U/ml penicillin, and 100 µg/ml streptomycin. The human teratocarcinoma cell line NTERA-2 (NT2) was obtained from DSMZ (Braunschweig, Germany) and cultivated on gelatin-coated dishes in DMEM supplemented with 10% FBS, 5% horse serum, 100 units/ml penicillin, and 100 µg/ml streptomycin. Differentiation of NT2 cells was induced with 10 µM retinoic acid (Sigma-Aldrich, St. Louis, MO, USA) as described elsewhere (Plotkin, 1984). Where indicated, the following reagents were added to the cell culture medium: aphidicolin (Sigma-Aldrich, final concentration: 5 µg/ml), hydroxyurea (Sigma-Aldrich, 1 mM), roscovitine (Millipore-Calbiochem, 5–50 µM), CVT313 (purchased as CDK2 inhibitor III from Millipore-Calbiochem, 10–50 µM), SU9516 (Santa Cruz Biotechnology, Santa Cruz, CA, USA, 2–10 µM), trichostatin A (Sigma-Aldrich, 100 ng/ml), MG132 (Sigma-Aldrich, 2.5 µM). To stop treatment, cells were washed several times with normal growth medium.

### Viruses

The HCMV strains AD169, Davis and Towne were purchased from ATCC (Manassas, VA, USA). Merlin and Toledo strains were a gift from Gavin Wilkinson (Cardiff, UK). The endotheliotropic isolates TB40/e and VHL/e were a gift from Christian Sinzger (Tübingen, Germany). All strains were grown on HEL fibroblasts. Virus titers were determined by IE1/IE2-fluorescence, essentially as described [68]. Briefly, quiescent HEL fibroblasts were infected with various dilutions of virus stocks. After 24 h of incubation, cells were fixed and stained with IE1/IE2-specific antibody. Subsequently, the number of positive cells was determined by flow cytometry and used to calculate viral titers. Unless otherwise stated, a multiplicity of infection (MOI) of 5 IE protein forming units (IEU) per cell was used for infection experiments. Where indicated, cells were pulse-labelled (60 min) with 10 µM 5-ethynyl-2'-desoxyuridine (EdU, Invitrogen, Carlsbad, CA, USA) before infection to label S phase cells. Non-incorporated EdU was removed by several washes with normal growth medium.

### Doxorubicin treatment and UV-irradiation

Doxorubicin (Sigma-Aldrich) was added to the cell culture medium to a final concentration of 1 µM. After two hours, cells were washed once with phosphate buffered saline (PBS) and fed with fresh culture medium. UV-irradiation was carried out using an UV-Stratalinker 2400 (Stratagene) equipped with 254 nm UV-light bulbs. For the duration of UV-exposure the culture medium was replaced with PBS (0.04 ml/cm^2^) to avoid the generation of toxic medium-derived photoproducts. A dosage of 10 J/m^2^ UV-C light was applied which leads in the HEL fibroblasts we used to high levels of p21 induction (data not shown).

### Flow cytometry

Cells were harvested by trypsinization, fixed and permeabilized by incubation in 75% ethanol for at least 12 h at 4°C and stained with specific antibodies and propidium iodide as described previously [13]. The following primary antibodies were used: anti-IE1/IE2 (clone E13, Argene, Verniolle, France), anti-gB (1-M-12, Santa Cruz), anti-pp28 (CH19, Santa Cruz), Oct3/4 (C-10, Santa Cruz). An Alexa Fluor 488-conjugated goat anti-mouse IgG antibody (Invitrogen) was used as secondary reagent. Isotype-specific antibodies were used for co-staining of IE1/2 (Alexa Fluor 546-conjugated goat anti-mouse IgG1_1_, Invitrogen) and Oct3/4 (Alexa Fluor 488-conjugated goat anti-mouse IgG1_2b_, Invitrogen). EdU-positive cells were detected using the Click-iT EdU Alexa Fluor 647 imaging kit (Invitrogen) according to the manufacturer's instructions. Cells were analyzed on FACScan or FACSCanto2 flow cytometers (BD Biosciences, San Jose, CA, USA) using CellQuest and FACSDiva software packages respectively. Cell doublets and aggregates were gated out of analysis. All experiments were performed at least thrice and only representative results were shown.

### Immunoblot analysis

Cells were lysed by sonication in 50 mM Tris-Cl (pH 6.8)–2% sodium dodecyl sulfate–10% glycerol–1 mM dithiothreitol–2 µg/ml aprotinin–10 µg/ml leupeptin–1 µM pepstatin–0.1 mM Pefabloc. The lysates were clarified by centrifugation at 17,500×g and protein concentration was determined using the Bio-Rad DC protein assay (Bio-Rad Laboratories, Hercules, CA, USA). Lysates were then adjusted to equal protein concentration, supplemented with 100 mM dithiothreitol and bromophenol blue, and heated to 95°C for 3–5 min. Sodium dodecyl sulfate-polyacrylamide gel electrophoresis and immunoblotting were performed accorded to standard procedures. The following primary antibodies were used: anti-p53 (clone DO-1, Santa Cruz), anti-p21 (C-19, Santa Cruz), anti-Cyclin A2 (C-19, Santa Cruz), anti-GAPDH (mAbcam 9484, Abcam, Cambridge, UK). HRP conjugated goat anti-mouse-IgG and goat anti-rabbit IgG (both Santa Cruz) served as secondary antibodies.

### Immunofluorescence microscopy

NT2 cells were grown on glass coverslips and treated as described above. After harvest of the coverslips, cells were washed, fixed, permeabilized and immunostained exactly as described [38]. A mouse monoclonal antibody against pp71 (clone 2H10, kindly provided by Tom Shenk, Princeton, NJ, USA) and an Alexa Fluor-488- coupled goat anti-mouse IgG (Invitrogen) were used as primary and secondary reagents. Nuclei were counterstained by the use of 4′,6-diamidin-2-phenylindol (DAPI). Images were acquired by an Eclipse A1 laser scanning microscope using NIS-Elements software (Nikon Instruments, Tokyo, Japan). Equal microscope settings and exposure times were used to allow direct comparison between samples.
